# Characterization of the transcripts and protein isoforms for cytoplasmic polyadenylation element binding protein-3 (CPEB3) in the mouse retina

**DOI:** 10.1186/1471-2199-10-109

**Published:** 2009-12-14

**Authors:** Xiang-Ping Wang, Nigel GF Cooper

**Affiliations:** 1 Department of Anatomical Sciences and Neurobiology, Health Sciences Campus, 500 S. Preston Street, University of Louisville, Louisville, KY, USA; 2 Department of Ophthalmology and Visual Sciences, Health Sciences Campus, 301 E. Muhammad Ali Boulevard, University of Louisville, Louisville, KY, USA

## Abstract

**Background:**

Cytoplasmic polyadenylation element binding proteins (CPEBs) regulate translation by binding to regulatory motifs of defined mRNA targets. This translational mechanism has been shown to play a critical role in oocyte maturation, early development, and memory formation in the hippocampus. Little is known about the presence or functions of CPEBs in the retina. The purpose of the current study is to investigate the alternative splicing isoforms of a particular CPEB, CPEB3, based on current databases, and to characterize the expression of CPEB3 in the retina.

**Results:**

In this study, we have characterized CPEB3, whose putative role is to regulate the translation of GluR2 mRNA. We identify the presence of multiple alternative splicing isoforms of CPEB3 transcripts and proteins in the current databases. We report the presence of eight alternative splicing patterns of CPEB3, including a novel one, in the mouse retina. All but one of the patterns appear to be ubiquitous in 13 types of tissue examined. The relative abundance of the patterns in the retina is demonstrated. Experimentally, we show that CPEB3 expression is increased in a time-dependent manner during the course of postnatal development, and CPEB3 is localized mostly in the inner retina, including retinal ganglion cells.

**Conclusion:**

The level of CPEB3 was up-regulated in the retina during development. The presence of multiple CPEB3 isoforms indicates remarkable complexity in the regulation and function of CPEB3.

## Background

Translational regulation plays a major role in temporal and spatial gene expression in a wide variety of situations. Modification of translation initiation factors lead to global regulation that controls the translation of the transcriptome as a whole. Modification of regulatory factors specifically binding to mRNA motifs in the 3' or 5' untranslated regions (UTRs) can modulate the translation of defined groups of mRNAs [1]. Accumulated evidence now indicates that mRNA-specific regulatory factors exist as either multi-protein complexes, such as cytoplasmic polyadenylation element binding proteins (CPEBs) [2], or multi-proteins complexes containing a non-coding RNA (siRNA or miRNAs) [3]. We now know that mRNA-specific translational control is essential for many biological processes including development, differentiation, and nervous system plasticity. Reports on the existence of these translational control mechanisms have added another layer of complexity to our understanding of gene regulation but this has been little explored in the retina.

Cytoplasmic polyadenylation was first brought to light in the 1980s, for its role in boosting translation of quiescent maternal mRNAs during oocyte maturation when little transcription activity is present [4-6]. This emerging area has particular significance for the nervous system because it provides insight into the molecular underpinnings of synaptic plasticity. The existence of a cytoplasmic polyadenylation mediated control system became a subject of interest to neuroscientists about a decade ago when it was first investigated in the hippocampus and the visual cortex [7]. In this case, CPEB1 was shown to control the polyadenylation and translation of Ca^2+^/calmodulin-dependent protein kinases α (CaMKIIα) mRNA upon N-methyl-D-aspartate receptor (NMDAR) activation. Four paralogous CPEBs (CPEB1-4) have been characterized in mouse [2,8,9]. One of these paralogs, CPEB3, is dendritically localized in the hippocampus and was shown to be co-immunoprecipitated with glutamate receptor subunit 2 (GluR2) mRNA. The knockdown of CPEB3 mRNA with the aid of small interfering RNAs (siRNA) resulted in enhanced translation of the synaptic protein GluR2 in neurons of the hippocampus [10].

Activity-dependent synaptic plasticity refers to the ability of neurons to change their synaptic strength and efficacy in adaptation to input. It can be embodied in several forms, including changes in the amount of neurotransmitters released from presynaptic terminals [11,12], alteration in the composition, density or activity of receptors/ion channels on postsynaptic membrane [13], re-remodeling of synaptic structure [14], and an increase or decrease in the number of synapses [15]. Synaptic plasticity has long been recognized at higher levels of the central nervous system (CNS), such as the cerebral cortex [16], the hippocampus [17], the cerebellum [18], and higher levels of the visual system [19].

Recent studies of the neural retina indicate that it may share some of these characteristics of activity-dependent plasticity. For example, dark-rearing suppressed the maturational pruning of dendrites in the inner plexiform layer which normally occurs after eye-opening [20-22]. Visual deprivation elevated the expression of several synaptic related molecules in the retina [23,24]. Light responsiveness and oscillatory potentials were inhibited in both young and adult dark-reared animals [25]. The composition of α-amino-3-hydroxy-5-methyl-4-isoxazole propionate receptor (AMPAR) in the retinal ganglion cells switches from predominantly GluR2-containing in the light phase to GluR2-lacking in the dark phase [26,27]. The molecular mechanisms controlling such events in the retina remain to be determined. A good candidate is CPEB-regulated translational control. Evidence has suggested the presence of CPEB1 in mouse retina [28], octpus retina [29], and the expression of CPEB1-4 mRNAs in embryonic Xenopus retina [30]. The current study demonstrates the existence of multiple CPEB3 transcript variants in the retina. The presence of CPEB3 protein is also shown in this tissue.

## Results

### Bioinformatics analysis of transcripts and protein isoforms of CPEB3

**Figure 1:**
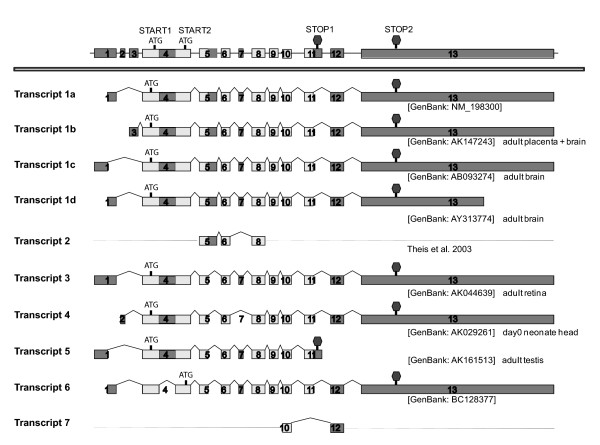
**Known transcripts of CPEB3**. Upper Panel: Representation of genomic DNA sequence. Boxes represented exons, and horizontal lines represented introns. Lower Panel: CPEB3 transcripts derived from alternative splicing. Ten transcripts were shown, with their accession numbers and types of tissue (if reported to the UniGene database) given to the right. The alternative splicings of exon 4, 5, 7 and 11 would generate different proteins products. The alternative splicings upstream of the start codon (exon 1-3) or downstream of the stop codon (exon 13) would give rise to different 5' and 3' UTRs, respectively. Dashed lines represented undetermined sequences. Partial sequence of transcript 2 (exon 5-7) was confirmed with the aid of PCR in a previous study [10], but its complete sequence was not documented in the UniGene database. Transcript 7 was a novel variant identified in the current study, of which the sequence upstream of exon 10 and downstream of exon 12 was not determined. Translational start codons and stop codons were annotated on top of the genomic DNA. Darkened boxed represented exons that could be alternatively spliced.

**Figure 2:**
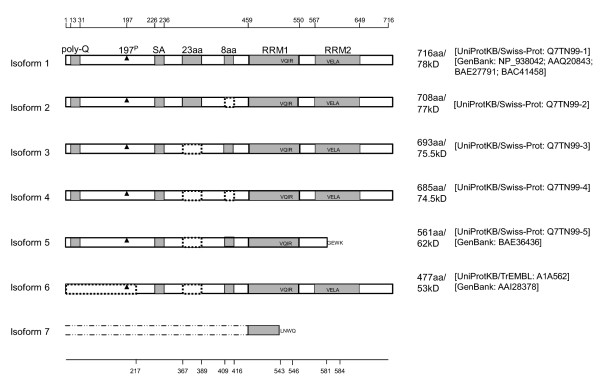
**Known isoforms of CPEB3 proteins**. Gray boxes represented possible functional motifs. Dash-lined boxed represented deletions. For simplicity, the names of the motifs were only labeled in isoform 1. Poly-Q (poly-glutamine) and SA (poly serine-alanine) were two unique motifs of CPEB3 that were absent in the other three CPEBs. In the current study, each of the seven isoforms was named corresponding to the same numbered cDNA transcripts depicted in figure 1. Protein isoform 1 was the longest (716 aa) and may be derived from cDNA transcripts 1a-1d. One possible polymorphorism was located at the 372^th ^amino acid of CPEB3 isoform 1: it was an N (asparagine) in [UniProtKB/Swiss-Prot: Q7TN99-Q7TN101] and [GenBank: AAQ20843] but a P (proline) in [GenBank: NP_938042, BAE27791, BAC41458]. Although the sequence of [GenBank: BAC41458] was reported as 722 aa, we presume it was 716 aa, since the first 6 amino acids QQAAQT preceding the 7^th ^amino acid M (methionine) was likely to be falsely generated by the prediction software. Isoform 7 was derived from computational translation based on the novel transcript 7 identified in the current study. The upstream sequence of this isoform was yet to be determined; but the in-frame translation of the identified sequence would result in truncated RRM1 ending with four unique amino acids (LNWQ) and removal of RRM2. 197^P ^represented the 197^th ^serine, a phosphorylation site [31]. The positions of the gray-box and dash-lined-box motifs were indicated at the top and the bottom, respectively. The lengths, molecular sizes and accession numbers were indicated to the right.

A previous study characterized four different alternative splicing variants of CPEB3 transcripts [9], in which two short motifs of 69 nucleotides (nt) and 24 nt (coding for 23 amino acids and 8 amino acids, respectively) are removed individually, or concurrently. However, the structure of CPEB3 seems to be more variable than previously reported. When investigating a nucleotide database (UniGene) for CPEB3 variants, we were able to identify eight transcripts labeled here as transcript variants 1a, 1b, 1c, 1d, 3, 4, 5 and 6 (figure 1). With the aid of sequence alignment tools (UCSC Genome Browser Blat), we mapped each cDNA to mouse genomic DNA, and acquired the information related to alternative splicing. In addition to the two previously reported short sequences (69 nt and 24 nt) whose presence or absence results in 4 variants [9], several other alternative splicing regions were discovered and elaborated here in diagrammatic form (figure 1). A similar search for the protein databases (UniProt and NCBI) identified six distinct CPEB3 protein isoforms labeled here as isoforms 1 through 6 (figure 2). With the bioinformatics tool Vector NTI, we inferred the matches between cDNA variants and protein isoforms, and designated the same numeric names to the matching transcripts and protein isoforms (figure 1, 2). Transcript 1a, 1b, 1c and 1d all give rise to protein isoform 1.

In addition to the previous reported alternative splicing regions [9], one of the alternative splicing locations identified in the current study was near exon 11. The prototypical transcripts have exon 12 immediately following exon 11 (figure 1, transcripts 1a-1d, 3, 4, and 6). However, transcript 5 has exon 11 extended, which causes a completely different 3' UTR and the omission of exon 12 (figure 1). As a result, the derived protein isoform 5 terminates with four unique amino acids (GEWK) and is devoid of the second RNA recognition motif (RRM) and the zinc finger domain (figure 2).

Another variable region identified here was located within exon 4. A 388 nt partial exon skipping within exon 4 causes the use of an alternative translation start codon (figure 1, transcript 6), resulting in the removal of the first 216 amino acids in the derived protein (figure 2, isoform 6). Interestingly, this deletion removed the 197th serine, which is a phosphorylation site of CPEB3 [31].

While the splicing patterns described above could result in various protein isoforms, we found several other splicing patterns which would not lead to altered protein sequences but would rather lead to variance in their UTR regulatory regions. For example, transcripts 1a, 1b, 1c use different 5'UTRs (exons 1, 2, and 3 respectively). Transcript 1d uses a different 3' UTR (via the use of an alternative polyadenylation signal in exon 13). Such variability is the result of differentially regulated transcriptional initiation and/or termination; it most likely leads to variation in the translational regulation of the specific transcripts. These interesting findings indicate the existence of a previously unrecognized complex regulatory control system for CPEB3 transcription and translation.

### Several variants including a novel variant of CPEB3 are identified experimentally in the retina. One of the variants is selectively expressed among different tissue-types

**Figure 3:**
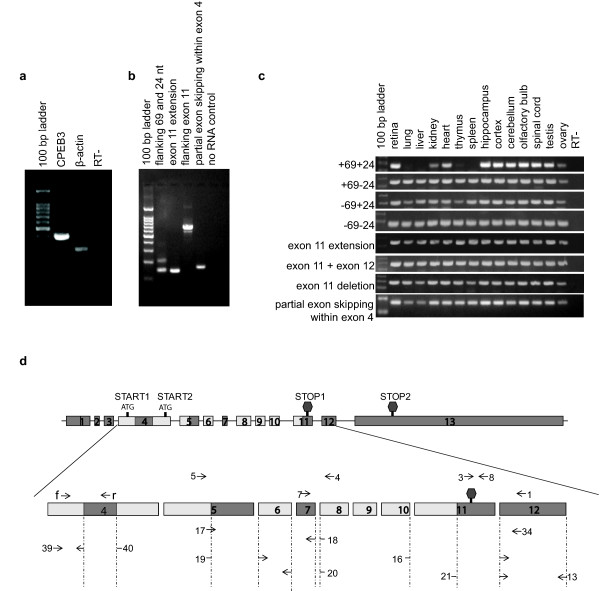
**The expression of CPEB3 as demonstrated by RT-PCR**. a) CPEB3 was present in the P60 retina. b) Multiple CPEB3 transcripts were present in the retina. Different primer sets for various CPEB3 transcripts (table1, table 2) were used for PCR. Each visible band was purified and sequenced, which confirmed the presence of six splicing patterns: "+69+24" (lane2, upper band), "-69+24" (lane2, lower band), "exon 11 extension" (lane3), "exon 11 + exon 12" (lane 4, upper band); a new pattern: "exon 11 deletion" (lane 4, lower band), and "partial exon skipping within exon 4". But it did not rule out the presence of "+69-24" and "-69-24", since the competition for the same set of primers by a dominant pattern ("-69+24") may mask the weakly expressed variants. c) Demonstration of "+69-24" and "-69-24" patterns and comparison of tissue distribution of all patterns. Primer sets specific for each individual splicing pattern were used for PCR on thirteen different tissues from adult mice. Each specific band was purified and sequenced for identity confirmation. The results demonstrated the presence of "+69-24" and "-69-24" patterns in the retina (lane 2) in addition to the 6 patterns identified in figure b). It also demonstrated the ubiquity of the majority of the patterns, with the exception of "+69+24", which was expressed in the CNS, the ovary, testis, kidney and heart, but not in the lung, liver, thymus and spleen. d) The locations of these primers were mapped to CPEB3. The corresponding relationships between the numeric primers and the splicing variants in figure a), b) and c) were listed in table 2. Darkened boxed represented exons that could be alternatively spliced.

We next investigated the expression of CPEB3 mRNA within the P60 mouse retina. Regular RT-PCR studies demonstrated that CPEB3 is present in the retina (figure 3a). We further investigated which of the known CPEB3 transcripts identified through our bioinformatics analyses are expressed in the retina. The primer sets for RT-PCR were designed to span exon 5-7, around exon 11, and the partial exon skipping site within exon 4 (figure 3d, table 1, table 2). Since one amplicon may correspond to more than one transcript (figure 1, figure 3d), for our purposes, we named each amplicon as an alternatively spliced "pattern" with a descriptive name rather than a numeric name. For example, the exon 5-7 region has four patterns ("+69+24", "-69+24", "+69-24", and "-69-24"), and the exon 11 region has two patterns ("exon 11 extension", or "exon 11 + exon 12"). The patterns within each splicing region are exclusive of one another. Each pattern correlated to one or multiple transcripts. The correspondence between each pattern and potential CPEB3 transcripts were listed in table 2.

**Table 1:** Primers used in RT-PCR.

Gene	Primer name	Primer sequence
β-actin	fwd	5'-TGAAGATCAAGATCATTGCTCC-3'
	rev	5'-TTAAAAAAACAAAGCCATGCC-3'
MAPK1	fwd	5'-GCATGGTTTGCTCTGCTTATGA-3'
MAPK1	rev	5'-ATTTTTATCTCTCTTAGGGTTCTTTGACAG-3'
CPEB3	fwd (f)	5'-CATCAAGGATAAACCGGTGC-3'
	rev (r)	5'-GAAGAATGGGGCAAACTTCC-3'
	#1	5'-TGTCAATGTCGTTGTGTTGAAGTTGC-3'
	#3	5'-GACTCCCACACCACAAGGATACACA-3'
	#4	5'-GAAGGCGTCTTCAAAGGGAAAGAGA-3'
	#5	5'-TATGATAAGGACTGACCATGAGCCTCTG-3'
	#7	5'-AGGAGCTATGGGCGGAGACGA-3'
	#8	5'-GCTGAGTCCCCAATGCCTTAGC-3'
	#13	5'-CCCGTTTGTCAATGTCGTTGTGTT-3'
	#16	5'-AAGGATAAACCGTTGAACTGGCA-3'
	#17	5'-CATGAGCCTCTGAAAGGTAAACACT-3'
	#18	5'-AAGACCGACCTCGTCTCCGC-3'
	#19	5'-CATGAGCCTCTGAAAGGACGC-3'
	#20	5'-GAAGACCGACTGTTAAGTGCCATA-3'
	#21	5'-TTCGAGCTGTTGAACTGGCA-3'
	#34	5'-CCGTTTGTCAATGTCGTTGTGT-3'
	#39	5'-TCTCAGCAGCAGCAGCGG-3'
	#40	5'-GCTGGGGCTGGCGGA-3'

**Table 2:** The splicing patterns of CPEB3, the corresponding CPEB3 primer pairs, and the associated transcripts.

Amplicon	Primer Pair	Corresponding Transcripts
CPEB3	f-r	
flanking 69 nt and 24 nt	4-5	
Flanking exon 11	1-7	
+69+24	17-18	transcripts 1a-1d
+69-24	17-20	transcript 2
-69+24	19-18	transcripts 3, 5, 6
-69-24	19-20	transcript 4
exon 11 extension	3-8	transcript 5
exon 11 + exon 12	21-13	transcripts 1a-1d, 3, 4, 6
exon 11 deletion	16-34	a novel transcript, transcript
partial exon skipping within exon 4	39-40	transcript 6

Amplicons of the PCR reactions were separated on agarose gel, and the identities of individual bands were confirmed by sequencing. Our results from these RT-PCR studies demonstrated the presence of "+69+24", "-69+24" (figure 3b, lane 2), "exon 11 extension" (figure 3b, lane3), the "exon 11 + exon 12" (figure 3b, lane 4 upper band), and "partial exon skipping within exon 4" splicing patterns in the retina. Additionally, a novel CPEB3 splicing pattern, "exon 11 deletion" (figure 3b, lane 4, lower band) was identified in the retina. This was the first time this latter pattern has been reported and was named here as "transcript 7" (figure 1).

In the exon 5-7 region, although only two bands were detected with RT-PCR, it is possible that more dominant transcripts "masked" weaker transcripts by competing for the same primers. Therefore, we designed separate sets of primers for each individual splicing pattern in this region to further confirm their presence or absence (figure 3c). Each specific band was purified and sequenced for confirmation. Our results demonstrated that the "+69-24" and "-69-24" splicing patterns were indeed present in the retina (figure 3c). Multiple tissues were used to determine the tissue specificity of the above splicing patterns. Of all the patterns examined, the majority did not appear to differ with respect to tissue distribution. The only exception was the "+69+24" pattern, which was expressed in the central nervous system, ovary, testis, kidney and heart, but was absent in the thymus, spleen, lung and liver (figure 3c).

### CPEB3 transcription is up-regulated during postnatal development of the retina

**Figure 4:**
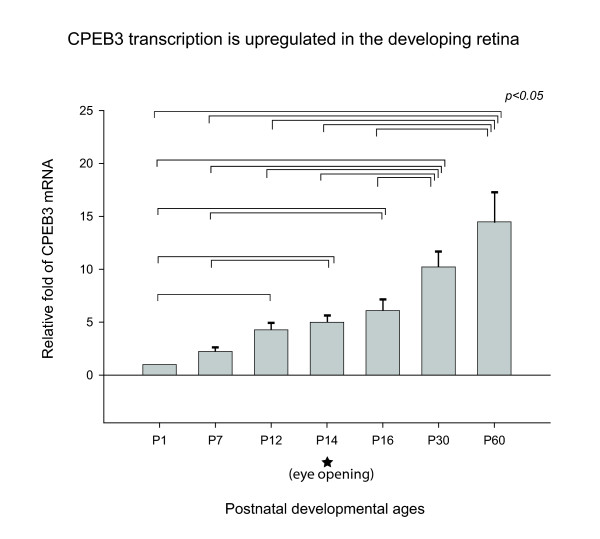
**CPEB3 mRNA during postnatal development**. The relative fold change in the amount of CPEB3 mRNA was shown with the aid of real-time PCR. Seven postnatal ages flanking the eye-opening event were used for this study. For each sample, the level of CPEB3 mRNA was normalized to that of 18S RNA in the exact same sample. All experiments were repeated three times. All ages were calibrated relative to the postnatal age P1 and expressed as fold changes. Statistically significant differences were found by ANOVA between sets of two bracketed ages as indicated (p <= 0.05). For each age, the number of samples n >= 6. Error bars represented standard error of the mean (SEM). The asterisk indicated the approximate time of eye-opening. The results demonstrated that CPEB3 was significantly up-regulated in the retina from P1 to P12 (before eye-opening), and from P16 to P30 (after eye opening), and reached maximum in the adulthood (P60).

To investigate the expression of CPEB3 during development, Taqman-based real-time PCR was used to evaluate the relative abundance of CPEB3 mRNA in the retinas of mice at seven different postnatal ages (figure 4). 18S RNA was used for normalization. An amplicon of 79 nt spanning exon 4 and exon 5 of CPEB3 was amplified. This amplicon was inclusive of all known CPEB3 transcripts, therefore, represented the totality of the CPEB3 transcripts. The similarity of the amplification efficiency of CPEB3 and 18S primer sets were pre-validated by the manufacturer and verified in our laboratory (data not shown). The level of CPEB3 mRNA was significantly increased throughout the postnatal development and stayed high when the animals reached adulthood (figure 4).

**Figure 5:**
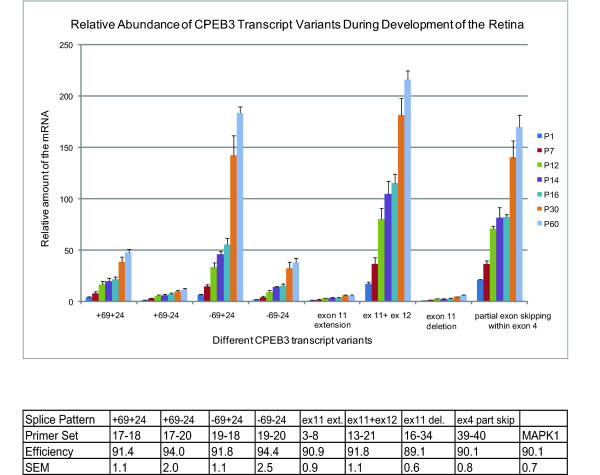
**Relative abundance of different CPEB3 transcript patterns during development of the retina**. The levels of each mRNA was normalized to the level of MAPK1 mRNA in the same sample. All the transcript variants demonstrated similar increasing trends during postnatal development. The relative abundance at any given age demonstrated that the "-69+24" pattern was the most abundant in exon 5-7 region; the "ex 11 + ex 12" pattern was the most abundant in the region surrounding exon 11; and "partial exon skipping within exon 4" pattern was abundant. For age P14, n = 3; for all the other ages, n = 4. Error bars represented standard error of the mean (SEM). The amplification efficiencies for all the primer sets used in realtime PCR were listed in the table below the graph.

Since CPEB3 has multiple transcript variants in the retina, we investigated which of the particular CPEB3 transcripts were up-regulated during the period of postnatal development using SYBR Green-based realtime PCR. Mitogen-activated protein kinase 1 (MAPK1) mRNA, the transcription level of which showed little fluctuation during development in the retina (data not shown), was used for normalization. MAPK1 primers and CPEB3 primer sets specific for each splicing pattern (table 1, table 2, figure 3d) were designed in-house to generate amplicons of between 75 bp and 150 bp for robust amplification. Similar amplification efficiencies (89%-94%) were reached under optimized conditions for all primers sets, allowing quantitative comparisons between CPEB3 and MAPK1, and among different CPEB3 splicing patterns (figure 5). The data on seven different age groups demonstrated that all CPEB3 transcript patterns examined had similar trends of postnatal-related increases (figure 5). This is consistent with our previous findings (figure 4).

Comparisons of the relative abundance of transcript patterns within each region (figure 5) demonstrated that within exon 5-7 region, the "-69+24" pattern was the most abundant, and the "+69-24" pattern was the least abundant. Within the region surrounding exon 11, the prototypical "exon 11 + exon 12" was the most abundant, while both "exon 11 extension" and "exon 11 deletion" were much less abundant. Of the eight transcript patterns, the three most abundant were "-69+24", "exon 11 + exon 12", and "partial exon skipping within exon 4" across all ages.

### CPEB3 protein is regulated during postnatal development of the retina

**Figure 6:**
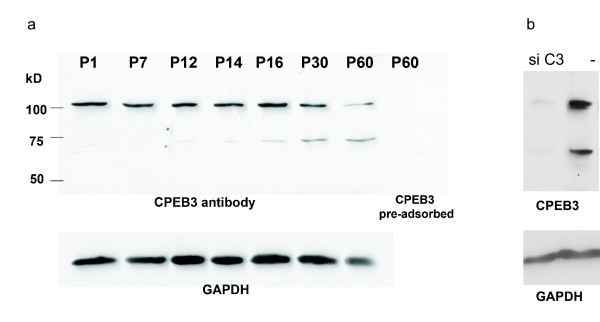
**The expression of CPEB3 proteins in the retina**. a) The regulation of CPEB3 protein during the postnatal development of the retina. CPEB3 western blot on retinal samples from seven different postnatal ages showed that the 75 kD band protein was increased during the development. Another band at ~130 kD was also present in the retina. Both bands diminished when the antibody was pre-adsorbed with the recombinant CPEB3 protein (the last lane). GAPDH was used as a loading control. b) Both of the 75 kD and the 130 kD bands diminished when Hela cells were transfected with CPEB3 siRNA. Cells in the negative control were treated with a negative control siRNA. GAPDH was used as a loading control.

We investigated the relative abundance of CPEB3 protein in the developing retina with the aid of western blots. Two antibody-labeled bands with molecular weights of ~130 kD and ~75 kD respectively were identified in western blots and were both diminished by pre-adsorption with recombinant CPEB3 protein or by depletion of CPEB3 in Hela cells (figure 6). The ~75 kD band could be isoform 3 or isoform 4 based on their predicted sizes. The intensity of the ~75 kD band increased significantly during postnatal development of the retina, which was consistent with the postnatal increases in the level of CPEB3 transcripts (figure 4, 5). The ~130 kD band could be a dimer, a pre-protein, or a prion form of CPEB3. Since the ~130 kD band was not weakened in reducing and denaturing conditions (by adding 1,4-dithioerythritol or beta-mercaptoethanol and boiling), it seems less likely to be a dimer. The possibility of it being a pre-protein needs further evidence. The transcripts shown here do not indicate the presence of a protein larger than ~78 kD, neither is there any such protein evident in the UniProt database. However, a previous study indicated the presence of a ~100 kD CPEB3 protein [10]. With the aid of a different gel/buffer system and different protein standards, we have since found that the larger band appeared to be around 97 kD. Therefore, it seems likely that the 100 kD band in the prior study and the 130 kD band in our study are of the same identity. We can only speculate that any transcripts for CPEB3 proteins larger than 78 kD are yet to be discovered. The possibility of it being a prion form [32,33] also needs further investigation.

Some of the transcripts identified in the current study did not show distinct protein bands in western blots. This may be attributed to the relative abundance of the transcript (a result of transcriptional regulation), or the variability in the 5' UTRs and 3' UTRs of the transcripts (leading to translational regulation). It is likely that not all transcripts are translated with equal efficiency under the current paradigm. Therefore, some protein isoforms may be expressed at very limited amounts, which are below the level of detection with the method used here. However, such isoforms may be expressed more robustly under other more appropriate, but unknown stimuli.

### CPEB3 transcripts are expressed in the inner retina

**Figure 7:**
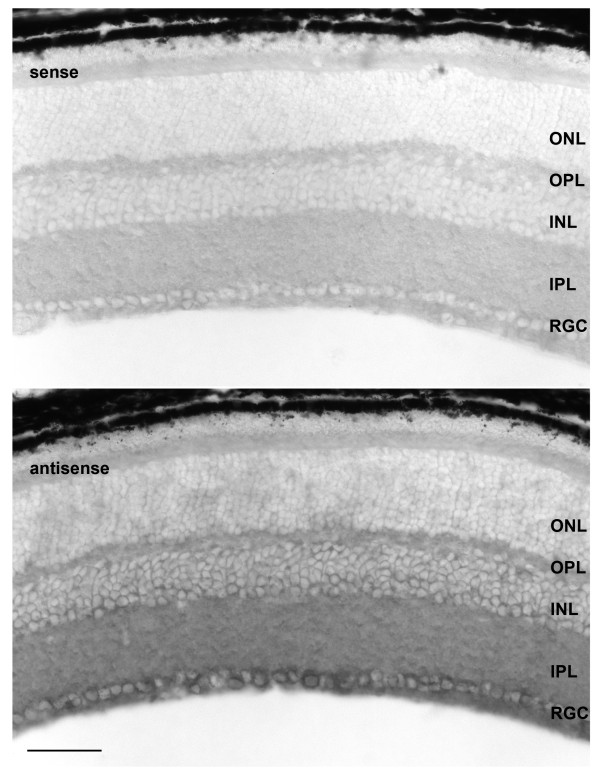
***In situ *hybridization of CPEB3 on P60 mouse retina**. Sense probe complementary to the antisense probe was used for the negative control. The probes were designed in a region with no significant homology to any other CPEB to ensure specificity. The expression of CPEB3 mRNA was located mostly in the RGC layer and to a lesser extent, the INL. Scale bar represented 50 μm. ONL: outer nuclear layer; OPL: outer plexiform layer; INL: inner nuclear layer; IPL: inner plexiform layer; RGC: retinal ganglion cell layer.

With the aid of *in situ *hybridization and light microscopy we identified which types of cells in the retina express CPEB3 mRNA in the P60 mouse. To ensure specificity, the *in situ *hybridization probes were designed in a region where little homology was found between CPEB3 and the other CPEBs. A sense probe complementary to the antisense probe was used as a negative control. The results (figure 7) demonstrated that CPEB3 was localized predominantly in the retinal ganglion cell (RGC) layer, and to a less extent, in the inner margin of the inner nuclear layer (INL). It was not clear if CPEB3 mRNA was also present in the plexiform layers due to the limited resolution of this technique.

### CPEB3 protein is expressed in the retina

**Figure 8:**
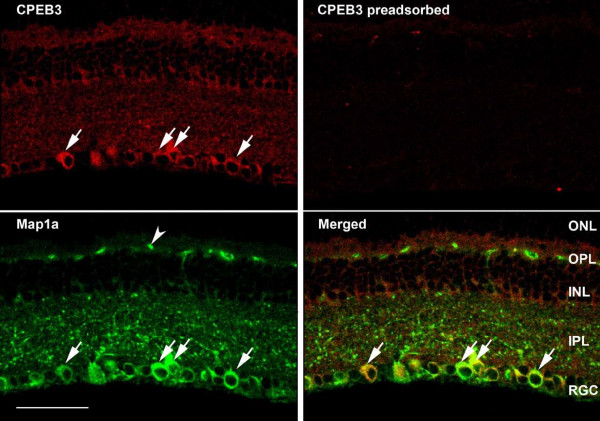
**The presence of CPEB3 in P60 mouse retina**. We used double-immunofluorescence labeling with antibodies to CPEB3 and Map1a. Map1a has been demonstrated as a marker for retinal ganglion cells. Most cells in the RGC layer were double-labeled with CPEB3 and Map1a antibodies (arrows), suggesting that they are retinal ganglion cells. The INL was also labeled with CPEB3 antibody, with the innermost cells more intensely labeled. Both the IPL and the OPL were labeled with CPEB3 antibody. The Map1a containing structures (arrowhead) in the OPL were non-specific since they also appeared in no primary control. The scale bar represented 50 μm.

**Figure 9:**
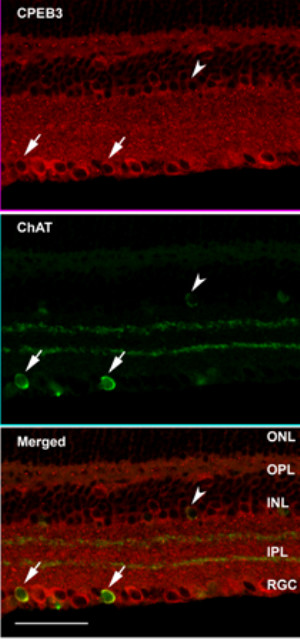
**The presence of CPEB3 in P60 mouse retina**. We used double-immunofluorescence labeling with antibodies to CPEB3 and ChAT. ChAT is a marker for cholinergic amacrine cells. A few cells in the RGC layer were double-labeled with ChAT and CPEB3 antibodies, as indicated with the arrows. This suggested that a few CPEB3 positive cells in the RGC layer were displaced amacrine cells. The size of such cells was usually smaller than those cells staining positive for both CPEB3 and Map1a. The INL was also labeled with CPEB3, with the innermost cells more intensely labeled. A few cells in the INL appeared to be double-immunopositive for ChAT and CPEB3 (arrowhead), suggesting that some CPEB3 positive cells in the INL were cholinergic amacrine cells. Both the IPL and the OPL were labeled with CPEB3. Scale bar represented 50 μm.

To confirm the expression pattern of CPEB3 mRNA and to better define the cell types containing CPEB3 protein, fluorescence immunocytochemistry with an antibody to CPEB3 was used. A pattern was observed which was similar to that seen for the localization by *in situ *hybridization. CPEB3 protein was strongly expressed in cells located within the RGC layer, and to a less extent, within cells of the inner boundary of the INL (figure 8, 9). Both the inner plexiform layer (IPL) and the outer plexiform layer (OPL) also showed pronounced immunolabeling of CPEB3. To define the type of cells which express CPEB3 we used double immunolabeling with cell type-specific markers in combination with CPEB3. The ganglion cell-specific marker microtubule-associated protein 1a (Map1a) [34,35], and the cholinergic amacrine cell marker choline acetyl transferase (ChAT) [36-41], were used. Confocal microscope images indicated that most of the anti-CPEB3 labeled cells in the RGC layer were retinal ganglion cells (figure 8), and a few were displaced amacrine cells (figure 9).

## Discussion

This study demonstrated for the first time that CPEB3 mRNA and protein were present in the mouse retina. Multiple CPEB3 transcripts were shown to be present in the retina. The majority of the transcripts described here appeared to have a wide tissue distribution. A novel transcript of CPEB3 ("exon 11 deletion") was discovered and was present in multiple tissues including the retina. In addition, the "+69+24" transcript pattern had various tissue distribution and was expressed in CNS tissues. The results also demonstrated for the first time that all CPEB3 transcripts were up-regulated in the mouse retina during postnatal development and reached maximum in adulthood.

### CPEB3 variants provide for a higher level of regulation complexity than has been hitherto recognized

The large number of variants in the coding region, together with variances in the 5' and 3' UTRs of CPEB3 (figure 1) provides evidence for an unexpected level of regulatory and functional complexity of CPEB3. First of all, the differences in CPEB3 protein sequences (figure 2) add to the complexity of its function which may involve different temporal and spatial patterns. The alternatively used sequences may generate discrete "tags" that serve as hallmarks for localization of the protein, catalysis by discrete kinases/phosphatases/endopeptidases, or interaction with different protein partners. As a result, the activation, inhibition or turnover of CPEB3 proteins may also be affected. For instance, the 8-amino acids motif (present in isoform 1, 3, 5, and 6, figure 2) presumably harbors phosphorylation sites for CaMKII, protein kinase A (PKA) and p70S6 kinases [9]. The functional significance of the 23-amino acids motif (present in isoform 1 and 2, figure 2) and the 4-amino acids motif GEWK (present only in isoform 5, figure 2) is unknown, but is of interest for future studies. The RNA recognition motifs (RRMs) of isoforms 5 and 7 of CPEB3 were altered, which would likely to impact their substrate specificities, similar to an effect previously proposed for CPEB1 [42].

Secondly, the regulatory properties of CPEB3 can stem from the differences in the 5' UTR and 3' UTR regions of the CPEB3 mRNAs. For example, transcripts 1a, 1b, 1c, and 1d all yield the same protein product, but each has a different 5' UTR or 3' UTR. The variability in the UTRs is the result of differentially regulated transcription (alternative transcriptional initiation and termination); such variability in the UTRs would further lead to discriminations in translational regulation. The UTRs may also harbor signal motifs such as that for mRNA localization or degradation, hence subjecting the mRNA to distinct stimuli or degradation pathways. The mechanisms for the transcriptional and translational control of CPEB3 and the functional significance of the UTR variants shown here provide fertile ground for future investigations.

### CPEB3 may play a role in synaptogenesis before eye opening

The level of CPEB3 was up-regulated during the postnatal development and reached maximum abundance in adulthood (figure 4, 5). The presence of CPEB3 in the early ages may be related to a possible role during synaptogenesis. It is well established that the mammalian retina is premature at birth. The ganglion cell layer and the inner plexiform layer are recognizable at the time of birth, but the remainder of the retina is yet to be fully developed. The synaptogenesis in the IPL starts at day 3 [43,44]. Robust glutamatergic synaptic formation occurs in the second postnatal week, and synaptic sublamina becomes visible in the IPL on day 12 [45]. The rate of synaptogenesis in the IPL drops precipitously at day 15 (around the time of eye-opening) [44]. Synaptic connections in the OPL begin to develop on day 3 and are well established by day 12, when the first light response was recorded as electroretinogram (ERG) [46]. The synaptogenesis in both the IPL and the OPL is concurrent with the increasing abundance of CPEB3 seen during the early ages. Also noteworthy is the pronounced labeling of CPEB3 proteins in the IPL and the OPL (figure 8 and 9). This prompts the speculation that CPEB3 expression is important for the process of synaptogenesis; or alternatively, its expression is a consequence of synapse formation.

Future studies on the interactions between CPEB3 protein and mRNAs for synaptically relevant molecules, such as AMPARs, NMDARs, CaMKII, and brain-derived neurotrophic factor (BDNF) mRNAs, can be employed to clarify this issue. For example, AMPAR is involved in fast synaptic transmission. GluR2/3 and 4 are expressed before the emergence of synaptic transmission, indicating a role of AMPAR in the establishment of retinal circuitry [47,48]. The onset and regulation of GluR2 expression in the retina is in accordance with the process of synaptogenesis: it starts early in postnatal development, and reaches a peak before eye-opening [48,49]. The distribution pattern of GluR2 in the IPL alters from a disoriented pattern just after birth to a laminated pattern prior to eye-opening [49], manifesting the reorganization of synaptic circuits. The expression pattern of CPEB3 in the retina observed in our study does appear to be consistent with that of GluR2. Thus, both are expressed in the same types of cells, and both are up-regulated in the first two weeks. Given the previous notion that CPEB3 protein binds to GluR2 mRNA in hippocampal neurons and that GluR2 is a potential target of CPEB3-mediated translational regulation [10], it is plausible to conceive that CPEB3 also regulates the translation of GluR2 during synaptogenesis in the retina.

In addition to GluR2, there are several other synaptically-relevant molecules that are localized in the RGCs and amacrine cells and are developmentally up-regulated, among which are AMPAR subunits GluR1, GluR4, NMDAR subunits NR1, NR2A, NR2B, and CaMKIIα [23,24,47,48]. A U-rich hairpin loop structure in the 3' UTR of mRNAs has been identified as the binding motif for CPEB4; such a motif was also recognized by CPEB3 [10]. Although other possible recognition motifs for CPEB3 protein remain to be identified, the presence of the U-rich hairpin structure in the 3' UTR of mRNAs could serve as an indicator that these mRNAs may be targets of CPEB3 regulated translational control. Potential CPEB3 targets containing the U-rich motif can be explored with bioinformatics tools, and further verified with *in vitro *binding assays [10] or *in vivo *manipulation of CPEB3 expression.

### CPEB3 may play a role during synaptic maturation

Anatomical studies indicated that the retina matures at postnatal day 41 [50]. Although most morphological and neurochemical properties of the mouse retina (i.e. the number of conventional and ribbon synapses, the expression of neurotransmitters receptors) have reached the adult level at the time of eye opening [43,51], the maturation of synaptic function continues for several weeks following eye-opening. The establishment of ON and OFF pathways occurs to a large extent after eye opening. The dendrites of RGCs ramifies widely in IPL during early postnatal days [52], but remodels into a predominantly monostratified form within sublamina a or sublamina b of the IPL in two to three weeks after eye opening [22]. The synaptic strength of the RGCs, measured as the frequency of spontaneous synaptic activity, is low around the time of eye opening, but surges around P25 both in spontaneous excitatory postsynaptic currents (sEPSCs) mediated by glutamate receptors and in spontaneous inhibitory postsynaptic currents (sIPSCs) mediated by GABA/glycine receptors [53]. These maturational processes following eye-opening resonate with the continuing increase of CPEB3 after eye opening. Whether such resonance is coincidental or interrelated is an intriguing topic for future studies.

### CPEB3 and synaptic plasticity in the adulthood

Both our real-time PCR data and western blot data indicated that CPEB3 continued to increase during development and reached its highest level in adulthood. These observations provide a strong indication that CPEB3 is functional in adult retinas. Neuronal plasticity has been historically characterized as a feature of the developing CNS, but several lines of evidence indicate that some forms of synaptic plasticity are not just the privilege of developing animals.

One form of activity-driven synaptic plasticity in the adult retina is described in the course of normal diurnal light/dark cycles. GluR2-containing AMPARs undergo rapid cycling in the RGCs and amacrine cells via endocytosis and exocytosis [26]. Synaptic quiescence in the dark drives rapid cycling resulting in the presence of AMPARs-lacking GluR2 in the synaptic membrane. On the contrary, synaptic activity in the light period inhibits cycling and stabilizes AMPAR as predominantly GluR2-containing receptors on the cell surface. Such cycling of AMPARs is also present in hippocampal neurons and plays a critical role in synaptic plasticity in the adult hippocampus [54,55]. Although the underlying mechanisms for AMPAR cycling are yet to be fully deciphered, one may speculate that CPEB3 plays a role in this process.

## Conclusion

In conclusion, the current study demonstrated that CPEB3 is expressed in the retina. The levels of all transcripts were up-regulated during postnatal development. We assume that all of these transcripts have corresponding protein products, but under the developmental paradigm used here, not all protein products were detected. It is possible that there are other paradigms which would lead to the translation of the other transcripts. This needs to be further explored. The variability in the protein-coding regions and the UTRs indicates a remarkable degree of complexity in both the regulation and function of CPEB3.

## Methods

### Bioinformatics

For CPEB3 transcript variants, the UniGene database [56] and the UCSC Genome Browser database [57] was used. For CPEB3 protein isoforms, the UCSC mouse proteome database [58], the UniProt database [59] and the NCBI protein database [60] were used. Sequence alignments were carried out with the aid of Blast [61], UCSC Blat [62], and Vector NTI (Invitrogen, Carlsbad, CA).

### Animal handling and tissue preparation

All animal experimental procedures were performed in compliance with University of Louisville IACUC animal care regulations, as well as the ARVO statement for the use of animals in ophthalmic and vision research. C57/BL6 mice were used for this study (Charles River Laboratories, Davis, CA). Mice were euthanized with CO2 followed by cervical dislocation. For RNA and protein extraction, the eyes were removed and retina dissected immediately and frozen on dry ice before proceeding to extraction. For *in situ *hybridization and/or immunohistochemistry, whole eyes were fixed in 4% paraformaldehyde for 12 hours at 4°C, then dehydrated in PBS buffer with 30% sucrose for two weeks at 4°C. Snap-freezing embedding was carried out in isopentane in a container placed on dry ice. The temperature was kept below -70°C by adding crashed dry ice into isopentane. The mounted tissue was then cut 14 microns thick on a cryostat on the same day or frozen air-tight at -80°C for future sectioning.

### RNA extraction

Frozen retina was homogenized rapidly using a PowerGen 250 homogenizer (Fisher Scientific, Pittsburgh, PA). Total RNA was extracted using RNeasy mini kit (Qiagen, Valencia, CA) following the manufacturer's instructions. The concentration of RNA was determined using a BioPhotometer (Eppendorf, Westbury, NY), and the quality of RNA decided by the ratio of 28S/18S on an agarose gel. RNA was frozen in -80°C for long term storage.

### RT-PCR

0.2 μg of total RNA was used for a 20 μl RT reaction using AMV reverse transcriptase (Promega, Madison, WI). 1 μl of the cDNA was used for PCR. The exon-exon junction spanning, gene-specific primers for CPEB3, the control gene β-actin, and specific CPEB3 transcripts were designed in-house and obtained from IDT (Coralville, IA). Sequences of these primers are listed in table 1. PCR was carried out on a thermocycler using the following conditions: 95°C 15 min for initial activation; 40 cycles of: 94°C 30 sec (denaturation), 60°C or 55°C 30 sec (annealing), 72°C 30 sec (extension); lastly, 72°C 10 min for extension. The resulting PCR products were visualized on 1% agarose gels and photographed. Individual bands were cut out, purified and sequenced to confirm their identities.

### Real-time PCR

0.2 μg of total RNA and the high capacity cDNA archive kit (Applied Biosystems, Foster City, CA) was used for the reverse transcription. The resulting cDNA was diluted 1:20 and 5 μl of the dilution was used for each PCR reaction. For the overall expression of CPEB3 during development, TaqMan Gene Expression Assay for CPEB3 (the mixture of gene-specific primers and gene-specific, FAM-labeled probes, ID# Mm01204293_m1, Applied Biosystems) and TaqMan Gene Expression Assay for the endogenous control gene 18S (ID# Hs99999901_s1, Applied Biosystems) were used. The CPEB3 primer-probe set generates a product spanning exon 4-5, which represents all known CPEB3 transcripts. PCR master mix containing Taq DNA polymerase, dNTPs and optimized reaction buffer were also obtained from Applied Biosystems. PCR reactions were performed with an ABI 7300 real-time PCR system using the following conditions: 50°C, 2 min; 95°C, 10 min; 40 cycles of (95°C, 15 sec; 60°C, 1 min). Data were analyzed with the aid of SDS2.1 software (Applied Biosystems). The RNA quantity for each sample was normalized to 18S in the same sample and then calibrated to P1. This provided an estimate of relative fold change compared to P1.

For the quantification of individual CPEB3 transcript variants, SYBR Green-based real-time PCR was used. MAPK1 was used for normalization. Transcript-specific, exon-spanning primer sets were designed in house (table 1) using Vector NTI; PCR master mix containing the DNA polymerase, dNTPs, optimized buffer, and SYBR Green was obtained from Applied Biosystems. PCR reactions were performed with an ABI 7300 real-time PCR system under the following conditions: 95°C, 10 min; 40 cycles of (95°C, 15 sec; 60°C, 1 min); 95°C, 15 sec; 60°C, 30 sec; 95°C, 15 sec. Inclusion of the dissociation step would reveal nonspecific products and primer dimmer or duplexes, if any. When the specificity was validated, the following optimization steps were performed for each set of primers: the amount of forward/reverse primers (ranging from 50 nM/50 nM to 900 nM/900 nM); the amount of cDNA (varying the dilution factors); the annealing temperature. Serial dilutions of a standard cDNA were used to generate standard curves, based which the amplification efficiencies were derived. Similarity within 5% difference was considered acceptable for quantitative comparisons. The primers were then used in quantitative analysis of developmental samples. The relative quantity of RNA for CPEB3 was normalized to that of MAPK1 in the same sample. Data were analyzed with the aid of SDS2.1 software.

### In situ hybridization

14 um frozen sections were used for *in situ *hybridization. Primers used in PCR amplification to generate the template for probes (IDT, Coralville, IA) are listed in table 3. PCR products were visualized on agarose gel and the specific band was cut out and purified using QIAquick Gel Extraction Kit (Qiagen, Valencia, CA). The identity of the purified product was confirmed by sequencing analysis, which was later used as template for *in vitro *transcription to generate DIG-labeled RNA probes (Invitrogen, Carlsbad, CA). RNA probes were denatured at 85°C for 5 minutes then chilled on ice. Slides were treated with 0.1 M Rnase-free triethanolamine (TEA)-HCl pH 8.0 for 5 min, washed, and pre-hybridized at room temperature for an hour in hybridization buffer, which was then replaced with hybridization solution containing 100-200 ng/ml of RNA probes for overnight incubation at 65°C. On the second day, slides were washed in 0.2 × SSC several times then transferred to buffer B1 (0.1 M Tris, 0.5 M NaCl, pH7.5) for 5 minutes. Slides were then incubated in buffer B2 (buffer B1 with 10% heat-inactivated sheep serum) for 1 hour at room temp, after which buffer B2 was replaced with buffer B2 containing anti-DIG antibody (1:5000) for overnight incubation. On the third day, slides were washed, equilibrated in buffer B3 (0.1 M Tris, 0.1 M NaCl, 50 mM MgCl2, pH9.5), and colorization reaction was carried out in buffer B4 (buffer B3 with 20 μl/ml NBT/BCIP stock solution and 0.1% tween-20). Slides were then mounted in Mowiol mounting medium and the results were analyzed with the aid of a microscope.

**Table 3:** Primers used to amplify probes for *in situ *hybridization.

Probe	Forward primer	Reverse primer
CPEB3-antisense	5'ACAGAGCCAGCTGCGCAAACCA3'	5'**GTAATACGACTCACTATAGGG** GAAGGTGCCTCCGAAGACCG3'
CPEB3-sense	5'**GTAATACGACTCACTATAGGG **ACAGAGCCAGCTGCGCAAACCA3'	5'GAAGGTGCCTCCGAAGACCG3'

The sequence in bold is T7 promoter.

### Protein extraction

Frozen retina was homogenized with the aid of a sonicator (Biologics, Inc., Manassas, VA) in CelLytic MT cell lysis buffer (Sigma, St. Louis, MO) with 1% (v/v) protease inhibitor cocktail (Sigma). Samples were centrifuged at 1000 × g at 4°C for 10 minutes to remove tissue debris. Protein concentration in the supernatants was determined with the aid of the Bradford assay (Sigma, St. Louis, MO). The samples were then dispensed into aliquots and stored in -80°C.

### Western blots

25 μg of total protein from each sample was loaded for each lane in polyacrylamide electrophoresis (PAGE) and gels were subsequently transferred to polyvinylidene fluoride (PVDF) membrane. GAPDH was used as a loading control. Antibodies were used at the indicated dilutions to label the western blots (table 4). Protein standards covering the range of 10 kD to 250 kD (Bio-Rad, Hercules, CA) were used as markers to determine the relative molecular weights of antibody labeled protein bands in western blots. Enhanced chemiluminescence reagent plus (ECL plus, Thermo Fisher Scientific, Rockford, IL) was used to detect the signal in the western blots. For negative controls we used recombinant CPEB3 protein to pre-absorb the CPEB3 antibody.

**Table 4:** Antibodies, sources, and working concentrations used in western blots.

Antibodies	Catalog and Company	Species	Working Concentration
CPEB3, polyclonal	Ab10883 Abcam, Cambridge, MA	Rabbit	1:1000
GAPDH, monoclonal	Img-5019A Imgenex, San diego, CA	Mouse	1:5000
HRP conjugated secondary antibody, goat anti rabbit	AP187P Millipore, Bedford, MA	Goat	1:30,000
HRP conjugated secondary antibody, goat anti mouse	AP130P Millipore, Bedford, MA	Goat	1:30,000

To examine antibody specificity we used a recombinant CPEB3 protein to pre-adsorb the antibody. This served as a negative control. The recombinant protein was separated with electrophoresis and subjected to mass spectrometry (MS) for identity confirmation. The fusion protein was then purified and used as a blocking reagent for the CPEB3 antibody.

### Recombinant proteins

Full-length CPEB3 cDNA was cloned into pGEX2T vector with a glutathionine S-transferase (GST) tag at the 5' end (Keyclone, Cincinnati, OH). The protein was induced in E. coli B21 cells by adding 0.8 mM isopropyl β-D-1-thiogalactopyranoside (IPTG). Inclusion bodies were purified, solubilized with Inclusion Body Solubilization Reagent (Thermo Fisher Scientific, Rockford, IL), and dialyzed to physiological condition in a progressively reduced concentration of urea in Tris·HCl (Thermo Fisher Scientific). The recombinant protein was used for pre-adsorption of antibody for western immunoblots to demonstrate specificity of the antibody.

### Immunohistochemistry

14 μm frozen sections were used for immunohistochemistry. All steps were carried out at room temperature. Sections were treated with TBS (pH7.4) containing 0.05% Triton X-100 for 10 minutes before being blocked with 10% normal donkey serum in TBS for 1 hour. Sections were then incubated with the primary antibodies at the indicated concentration for 1 hour. After several washes, secondary antibodies were applied for 1 hour. Slides were washed again before being cover-slipped with Vectashield mounting medium (Vector Laboratories, Burlingame, CA). Immunolabeled sections were photographed with the aid of a confocal microscope (Olympus, La Jolla, CA) using 40× objective. A stack of 1 μm scans was combined to produce the final images. The antibodies used in this study, their source and dilutions are listed in table 5.

**Table 5:** Antibodies, sources, and working concentrations used in immunohistochemistry.

Antibodies	Catalog and Source	Species	Working Concentration
CPEB3, polyclonal	Ab10883 Abcam, Cambridge, MA	Rabbit	1:50
Map1a, monoclonal	M4278 Sigma,, St. Louis, MO	Mouse	1:200
ChAT, polyclonal	AP144-p Millipore, Bedford, MA	Goat	1:200
Alexa Fluor^® ^488 donkey anti-rabbit IgG (H+L)	A11034 Invitrogen, Carlsbad, CA	Donkey	1:200
Alexa Fluor^® ^594 donkey anti-mouse IgG	A21203 Invitrogen, Carlsbad, CA	Donkey	1:200
Alexa Fluor^® ^594 donkey anti-rabbit IgG (H+L)	A21207 Invitrogen, Carlsbad, CA	Donkey	1:200
Alexa Fluor^® ^488 donkey anti-goat IgG	A11055 Invitrogen, Carlsbad, CA	Donkey	1:200

### siRNA knockdown of CPEB3

Hela cells at confluence of 50-70% were transfected with 100 nM siRNAs duplexes using siPORT NeoFX reagent (Applied Biosystems, Foster City, CA) in serum-containing medium. Cells were incubated with siRNA-containing medium for 48 hours before being harvested. Proteins were extracted using CelLytic M Mammalian Cell Lysis/Extraction Reagent (Sigma, St. Louis, MO) with 100× protease inhibitors (Thermo Fisher Scientific, Rockford, IL) and used for western blots. The sequences of CPEB3 siRNA duplexes were as following: sense strand: CGAGUGGAACGCUACUCUAtt, antisense strand: UAGAGUAGCGUUCCACUCGtt. Silencer^® ^Negative Control #1 siRNA (Part Number AM4635, Applied Biosystems, Foster City, CA) was used as a negative control.

## Authors' contributions

This was in partial fulfillment of the PhD degree of XPW. XPW has contributed to the conception and design, the experiments, the acquisition and analysis of data, and the drafting and revision of the manuscript. NGFC has contributed to the conception and design, the supervision of this study, the analysis and interpretation of data, the revision and final approval of the manuscript. NFGC also acquired funding for this project. All authors read and approved the final manuscript.
